# Medical and non-medical complications among children and adolescents with excessive body weight

**DOI:** 10.1186/1471-2431-14-232

**Published:** 2014-09-14

**Authors:** Albane BR Maggio, Xavier E Martin, Catherine Saunders Gasser, Claudine Gal-Duding, Maurice Beghetti, Nathalie J Farpour-Lambert, Catherine Chamay-Weber

**Affiliations:** Pediatric sports medicine and obesity care program, Division of pediatric specialties, Department of Child and Adolescent, University Hospitals of Geneva and University of Geneva, 6, rue Willy-Donzé, 1211 Geneva 14, Geneva, Switzerland; Pediatric Cardiology Unit, Division of pediatric specialties, Department of Child and Adolescent, University Hospitals of Geneva and University of Geneva, Geneva, Switzerland

**Keywords:** Cardiovascular disease, Musculo-skeletal, Metabolism, Co-morbidities, Childhood obesity

## Abstract

**Background:**

The burden of disease from childhood obesity is considerable worldwide, as it is associated with several co-morbidities, such as dyslipidemia, hypertension, type 2 diabetes (T2DM), orthopedic and psychosocial problems. We aimed at determining the prevalence of these complications in a population of children and adolescents with body weight excess.

**Methods:**

This is a cohort study including 774 new patients (1.7 - 17.9 yrs, mean 11.1 ± 3.0) attending a pediatric obesity care center. We assessed personal and family medical histories, physical examination, systemic blood pressure, biochemical screening tests.

**Results:**

We found that the great majority of the children suffered from at least one medical complication. Orthopedic pathologies were the most frequent (54%), followed by metabolic (42%) and cardiovascular disturbances (31%). However, non-medical conditions related to well-being, such as bullying, psychological complaints, shortness of breath or abnormal sleeping patterns, were present in the vast majority of the children (79.4%). Family history of dyslipidemia tends to correlate with the child’s lipids disturbance (p = .053), and ischemic events or T2DM were correlated with cardiovascular risk factors present in the child (p = .046; p = .038, respectively).

**Conclusions:**

The vast majority of obese children suffer from medical and non-medical co-morbidities which must be actively screened. A positive family history for cardiovascular diseases or T2DM should be warning signs to perform further complementary tests. Furthermore, well-being related-complaints should not be underestimated as they were extremely frequent.

**Electronic supplementary material:**

The online version of this article (doi:10.1186/1471-2431-14-232) contains supplementary material, which is available to authorized users.

## Background

Children with body weight excess are an important high risk group for health complications. This population suffers already at an early age of multiple complications leading to increased health risks and costs in adult life [1–3].

However, there are disparities in epidemiological data, depending on where the studies are performed, the selected populations and the clinical settings. Indeed, most childhood obesity co-morbidities are rare in the general population, but are likely to be of increasing importance as even if stabilizing, the prevalence of childhood obesity in our country is still of 20% [4]. Therefore, it is important to collect data from a clinical population to evaluate their prevalence. To our knowledge, no studies have investigated the concomitant occurrence of medical (cardiovascular, metabolic, orthopedic) and non-medical complications in a large sample of overweight or obese children and adolescents. Therefore, we aimed first at determining the prevalence of obesity-related co-morbidities in a cohort of children with body weight excess consulting in a specialized obesity center and second at comparing those complications between overweight, obese and extremely obese subjects. We hypothesized that the numbers of complications increase with weight status.

## Methods

### Study design and subjects

This was a cohort study including 774 subjects with excessive body weight (BMI z-score above one SD for age and gender) [5], aged 1.7 to 17.9 years (mean age 11.1 ± 3.0), attending our Pediatric Obesity Care Center between January 2008 and August 2012. Our center is the only one taking care of obese children in our county for a population of 49’500 children aged between 5 and 16 years old. Data were collected both retrospectively and prospectively. Children were referred, if they had a BMI z-score above one SD, by their general practitioners, school nurses or by the Child and Adolescent Department of the Geneva University Hospitals to follow a multidisciplinary therapeutic program. The only exclusion criterion was a normal weight (BMI z-score under one SD).

Once informed, a written consent was obtained from both parent and child during the prospective phase of the study. All subjects accepted to participate. The Mother and Child Ethics Committee of the University Hospitals of Geneva approved the study.

### Measures

Medical history, physical examination and laboratory tests were used to define medical complications: orthopedic (analyzing 3 different complications); cardiovascular (analyzing 7 different risk factors) and metabolic (analyzing 4 different anomalies); as well as non-medical complications related to well-being (analyzing 4 different complaints).

### Medical history

At the first visit, a semi-structured interview (see Additional file 1) following an established protocol was taken by a pediatrician and used similarly for all subjects, to obtain a detailed personal medical history. We assessed the frequent complaints reported in childhood obesity that are known to have an impact on their well-being, such as bullying, psychological complaints (depressiveness/poor emotional feelings, preoccupation with physical appearance and self-esteem), shortness of breath or abnormal sleeping patterns (delayed onset or early or frequent awakenings). We had only 6% of missing data.

We also obtained a family medical history through parent report, including parents and grand-parents, searching for the presence of obesity, type 2 diabetes (T2DM), dyslipidemia, systemic hypertension and ischemic events, such as myocardial or cerebral infarctions. The ethnic background was classified depending on parental origin: European, Hispanic, Asian, African or Oriental.

### Physical examination

We measured body weight (kg), height (cm), and waist circumference (cm). Body mass index (BMI) was calculated as weight/height squared (kg·m^-2^) and z-scores were calculated using the World Health Organization references [5]. Children with a BMI z-score between one and two, between two and three and above three were defined as overweight, obese and extremely obese, respectively. During physical examination, we examined all subjects for acanthosis nigricans, and for signs of orthopedic conditions such as pes planus (the entire internal plantar arch comes into complete or near-complete contact with the ground), hyperlordosis and genu valgum (intermalleolar distance ≥ eight cm). We had only 6% of missing data for the orthopedic examination.

### Laboratory

Fasting plasma glucose [mmol·l^-1^], total cholesterol (TC), high-density lipoprotein cholesterol (HDL-C), triglycerides (TG) and alanine aminotransferase (ALAT) levels [U.l^-1^] were determined using standard automated techniques (SYNCHRON LX20^®^). Low-density lipoprotein cholesterol (LDL-C) level [U.l^-1^] was calculated according to the Friedewald formula [6]. Thyroid-stimulating hormone (TSH) [mUl.^-1^] was measured using an automated immunoassay analyzer platform and insulin concentration using radioimmunoassay (Access^®^ ultrasensitive insulin, Beckman Coulter Ireland Inc.). Insulin resistance was evaluated by the homeostasis model (HOMA-IR = fasting insulin [μU·ml^-1^] × fasting glucose [mmol·l^-1^]/22.5) and an oral glucose tolerance test was performed when needed [7].

Results were considered abnormal if: TC, LDL-C, and TG levels were > 95th percentile and HDL-C < 5th percentile for age and gender according to pediatric guidelines [8]; ALAT > 40 (U.l^-1^); TSH: > 4 (mUl.^-1^) [9]; insulin > 15 (μU·ml^-1^); HOMA-IR > 4 [10]. Impaired fasting glucose (IFG), impaired glucose tolerance (IGT) or type 2 diabetes (T2DM) were defined according to the American Diabetes Association [11].

Laboratory tests were considered in the analysis if performed in a time scale of two months around the first visit. In average we had 63% of missing data for the blood analysis.

### Blood pressure

Systemic blood pressure was measured on the right arm after 10 minutes’ rest in the supine position, using a standard, automated device (Philips SureSigns VS3, Philips Medical System, Andover, USA). The cuff covered two-thirds of the length of the upper arm, with the length of the bladder covering the arm circumference. In case of values above the 95th percentage for age, gender and height [12], measurements were taken again 3 times after 2 to 5 minutes and the lowest was recorded. Systolic and diastolic blood pressure z-scores were determined [13], and hypertension was defined as values above the 95th percentile for age, gender and height [12]. We had 13% of missing data.

There was no missing data in 174 patients.

### Statistical analysis

Statistical analyses were performed using the SPSS software 18.0 (Chicago, IL). Data were presented as mean and standard deviation (SD) or number and valid percentage. Statistical differences between overweight and obese subjects concerning occurrence of complications, age, BMI z-score and gender were analyzed using independent Student t-test or chi-square test. A multiple logistic regression model was used to estimate the odd ratio (OR) to have a selected complication between overweight (controls) and obese (cases) children, controlling for age and gender. Analysis of variance (ANOVA) with Bonferroni post-hoc test was used to compare the presence of complications per weight status, age categories and ethnic groups. We evaluated also the relationship between complications and BMI z-scores using linear regression. Pearson coefficient correlation was used to correlate family medical history and the presence of complications in the child. Differences were considered significant if P < 0.05.

## Results

### Subjects’ characteristics (n = 774)

There were 399 (52%) girls, mean age was 11.1 ± 3.0 years and mean BMI z-score was 2.7 ± 0.9. Our cohort was composed of 15.4% overweight subjects (n = 119/774; mean age: 12.1 ± 2.6 yrs), 56.3% obese (n = 436; mean age: 11.4 ± 2.6 yrs) and 28.3% extremely obese children (n = 219; mean age: 9.8 ± 3.6 yrs, representing 33.4% of the obese group).

Ethnic background showed that 46.8% came from European countries, 15.0% Hispanic, 16.9% African, 7.2% Oriental and 3.4% Asian countries. Data were missing for 10.7% (n = 83/774).

Table 1 presents the main obesity-related complications according to weight status, as well as the risks (OR) of these complications for obese or extremely obese children to present such conditions compared to overweight subjects, when controlling for age and gender.

**Table 1:** **Selected obesity-related complications according to weight status**

	Overweight	Obese	Extreme obese		
**N**	119	436	219		
**Age (years)**	12.1 ± 2.6	11.4 ± 2.6	9.8 ± 3.6		
**Gender (% girls)**	74	53	36		
**BMI z-score**	1.7 ± 0.24	2.5 ± 0.3	3.8 ± 0.8		
**WC (cm)**	78.3 ± 9.8	84.0 ± 11.2	87.4 ± 15.7		
	**n; (% within obesity status)**			**OR (95% CI)**	**OR (95% CI)**
				**Obese**	**Extreme Obese**
**Orthopedic complications:**					
**Genu valgum**	15/119 (12.6)	145/436 (33.3)	98/218 (45.0)	3.4 (1.9 – 6.2)***	4.7 (2.5 – 8.8)***
**Pes planus**	18/104 (17.3)	104/416 (25.0)	79/207 (38.2)	1.4 (0.8 – 2.5)	2.1 (1.1 – 3.9)*
**Hyperlordosis**	14/104 (13.5)	101/416 (24.3)	70/207 (33.8)	1.9 (1.0 – 3.6)*	2.3 (1.1 – 4.5)*
**Cardiovascular risk factors:**					
**Systolic HTN**	8/99 (8.1)	63/388 (16.2)	45/188 (23.9)	2.5 (1.2 – 5.5)*	4.8 (2.1 – 11.1)***
**Diastolic HTN**	2/99 (2)	23/387 (5.9)	16/186 (8.6)	3.1 (0.7 – 13.4)	3.9 (0.8 – 18.7)
**High TC**	11/31 (35.5)	26/158 (16.5)	17/101 (16.8)	0.27 (0.1 – 0.7)**	0.21 (0.1 – 0.6)**
**High LDL-C**	7/30 (23.3)	15/156 (9.6)	14/99 (14.1)	0.29 (0.1 – 0.8)*	0.37 (0.1 – 1.2)
**Low HDL-C**	3/30 (10)	31/157 (19.7)	31/100 (31.0)	2.3 (0.7 – 8.3)	4.4 (1.2 – 16.3)*
**Metabolic complications:**					
**IFG/IGT/T2DM**	5/31 (16.1)	19/162 (11.7)	8/103 (7.8)	0.83 (0.3 – 2.6)	0.62 (0.2 – 2.3)
**Hyperinsulinemia or IR**	11/27 (40.7)	40/141 (28.4)	35/91 (38.5)	0.81 (0.3 – 2.0)	2.2 (0.8 – 6.1)
**High ALAT**	2/19 (10.5)	8/108 (7.4)	15/77 (19.5)	0.48 (0.1 – 2.9)	1.6 (0.3 – 9.8)
**High TSH**	2/29 (6.9)	10/147 (6.8)	9/88 (10.2)	0.95 (0.2 – 4.7)	1.6 (0.3 – 8.7)
**Non-medical complications:**					
**Shortness of breath**	17/104 (16.3)	119/417 (28.5)	95/207 (45.9)	1.86 (1.1 – 3.3)**	3.15 (1.7 – 5.9)***
**Sleep disturbance**	10/104 (9.6)	45/416 (10.8)	38/207 (18.4)	1.06 (0.5 – 2.2)	1.62 (0.7 – 3.6)
**Bullying**	30/104 (28.8)	154/417 (36.9)	78/207 (37.7)	1.50 (0.9 – 2.4)	1.58 (0.9 – 2.7)
**Psychological complaints**	67/105 (63.8)	290/417 (69.5)	122/207 (58.9)	1.54 (0.9 – 2.5)	1.35 (0.8 – 2.3)

Data are express as mean and standard deviation.

For limits of pathological values, refer to the Methods section.

Odd ratio (OR) controlled for age and gender; controls being the overweight group.

*p < .05, **p < .01 and ***p < .001.

WC: waist circumference; HTN: hypertension; TC: total cholesterol; IFG: impaired fasting glucose; IGT: impaired glucose tolerance; T2DM: type 2 diabetes; IR: insulin resistance.

### Orthopedic complications

Orthopedic conditions were present in 53.6% of subjects and their prevalence increased with weight status (ANOVA with post-hoc test: p < .001 between each groups). Genu valgum was the most commonly found complication (33%, n = 258/773), followed by pes planus (28%, n = 201/727) and hyperlordosis (25%, n = 185/727).

### Cardiovascular risk factors

Cardiovascular risk factors, such as hypertension (HTN) and lipid anomalies, were present in 31.2% of subjects, and their global prevalence was significantly increased in the obese and extremely obese groups compared to the overweight subjects (p < .01). Systolic and diastolic HTN were present in 17% (n = 116/675) and 6% (n = 41/672) of screened children, respectively. They were simultaneously present in 4% of subjects (n = 25/672). Blood pressure z-scores were highly related to the BMI z-score (systolic: t = 5.0 and diastolic: t = 4.0; p < .001 for both). In fact, the risk to develop systolic HTN increased with the weight status, as when compared to overweight subjects, the risk increased of 2.5 for obese and almost five-fold for extremely obese children (Table 1).

Forty percent of children had dyslipidemia. Low HDL-C was the most frequent disturbance (23%, n = 65/287), followed by high TC (19%, n = 54/290) and LDL-C (13%, n = 36/285). High TG was very rare with only two obese and one extremely obese subjects being above the normal range. BMI z-score was higher in subjects with low HDL-C (3.2 ± 0.8 vs. 2.8 ± 0.8 U.l^-1^, p = .001) and TC and LDL-C levels were more frequently abnormal in overweight compared to obese children (Table 1).

### Metabolic complications

Metabolic complications, such as abnormal glucose, insulin, TSH or ALAT levels, were present in 42.1% of subjects, without difference among weight status (Table 1). Glucose metabolism was normal in almost 90% of children. After oral glucose tolerance tests, two of them (1%) were diagnosed with T2DM (one girl aged 13 yrs with a BMI z-score of 2.3 and a boy aged 16 yrs with a BMI z-score of 3.6), 4% of them had impaired glucose tolerance (n = 12/296; mean age: 13.3 ± 2.2 yrs; mean BMI z-score: 2.9 ± 0.8) and 6% of them had impaired fasting glucose (n = 18/296; mean age: 12.1 ± 3.0 yrs; mean BMI z-score: 2.6 ± 0.6). The rate of hyperinsulinemia or insulin resistance was high (33%, n = 86/259), but was independent of the weight status when controlled for age and gender. Furthermore, almost 20% (n = 144/727) presented an acanthosis nigricans on clinical examinations, which was positively correlated with insulin resistance for 44% (r = 0.132, p = .032), with a tendency for hyperinsulinemia in 38% of cases (r = 0.116, p = .059).

We also assessed TSH and ALAT concentrations in some children. We found elevated TSH and ALAT levels in 8% (n = 21/264) and 12% (n = 25/204) of them, respectively. There was no difference between weight status (Table 1); however, ALAT increased significantly with BMI z-score (t = 2.8, p = .006) and insulin concentration (t = 5.0, p < .001). ALAT was also more frequently abnormal in boys (21% of boys vs. 5% of girls; p = .001). Only one child and two adolescents had ALAT concentrations above 100 U.l^-1^ leading to immediate complementary tests, allowing other liver diseases to be ruled out. Those three subjects showed improved ALAT concentrations at follow-up. No subjects were diagnosed with liver diseases during their follow-up.

Waist circumference was a good indicator of metabolic disturbance, as it was significantly higher in children with abnormal glucose metabolism, hyperinsulinemia or high HOMA-IR (waist circumference with normal HOMA-IR: 81.8 ± 10.8 vs. with abnormal HOMA-IR: 91.5 ± 12.3; p < .001), even after adjusting the age.

### Non-medical complications

Non-medical complications related to well-being were assessed using a semi-structured interview during the clinical evaluation and concerned 79.6% of them. Shortness of breath during physical activities was reported by 32% (n = 231/728) of the subjects, especially in obese and extremely obese children (Table 1), and poor sleep quality was observed for 13% (n = 93/727) of them. Almost forty percent of them (n = 262/728) were victims of bullying at school or at home, and for 66% (n = 479/729) the physical appearance was a major issue, independently of their weight status or BMI z-score.

### Age role

**Figure 1:**
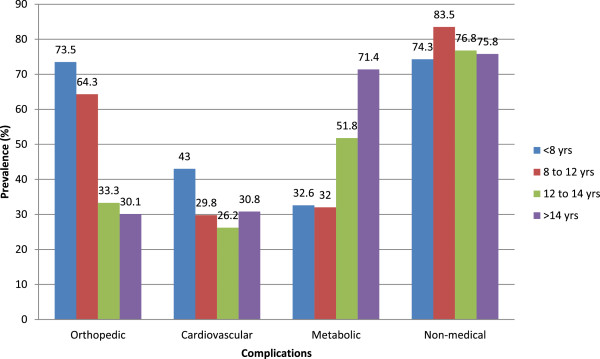
**Prevalence of medical and non-medical complications by age category.**

Children were also classified according to their age: less than 8 years old (n = 117/774, 15.1%), 8 to 12 (n = 349, 45.1%), 12 to 14 (n = 164, 21.2%) and more than 14 years old (n = 144, 18%).The percentage of medical and non-medical complications by age category is shown in Figure 1. The frequency of the three orthopedic conditions (hyperlordosis, pes planus and genu valgum) decreased progressively with age, being more frequent in children younger than 8 years old compared to the other age groups (p < .001 for all).

The rate of systolic and diastolic HTN were also higher in younger children (<8 yrs) (p < .05 for all), but there was no difference in lipids anomalies among age groups.

On the contrary, glucose, insulin and ALAT anomalies were more frequent in adolescents older than 14 years compared to the other age groups (p < .05 for all).

Poor sleep quality, as well as shortness of breath was more frequent in children younger than 12 years, but psychological complaints were less frequent in the younger ones (<8 years) (p < .05). There was no difference concerning bullying among age categories.

### Role of ethnicity

Ethnic differences were found only for metabolic complications (p = .002), with a lower prevalence in African compared to European (African: n = 12/55, 9.1% vs. European: n = 69/150, 19.1%; p = .036) or Asian subjects (Asian: n = 7/12, 26.9%; p = .003). Those differences concerned the insulin anomalies in relation to European (African: n = 6/51, 4.6% vs. European: n = 47/131, 13%; p = 0.013) and ALAT anomalies in relation to Asian (African: n = 0/41 vs. Asian: n = 5/11, 19.2%, p < .001). In fact, ALAT levels were abnormal more frequently in Asian compared to the other ethnic groups (European: n = 10/98, 2.8%, p = .003; Hispanic: n = 4/30, 3.4%, p = .026; Oriental: n = 2/15, 3.6%, p = .073).

### Family history

Family medical history revealed many complications in parents and grand-parents, as at least one of them suffered from: systemic HTN in 42% (n = 299/719), dyslipidemia in 35% (n = 250/719), T2DM in 13% (n = 73/560) and ischemic event (myocardial or cerebral) in 16% of cases (n = 120/771). Family dyslipidemia tended to be correlated to the number of lipid abnormalities found in the child (r = 0.115, p = .053), and history of ischemic event or T2DM were correlated with the total number of cardiovascular risk factors in the child (ischemic event: r = 0.074, p = .046; T2DM: r = 0.09, p = .038).

### Summary of complications in a sub-group of subjects

As to minimize the potential bias due to missing data, we calculated the prevalence of the complications, as well as their correlation with BMI z-score, in a sub-group of subjects without missing data (n = 174). Compared to the entire cohort, the gender repartition (52.9 vs. 52% of girls), as well as mean ages and BMI z-scores (mean ages: 10.6 ± 2.9 vs. 11.1 ± 3.0 yrs; mean BMI z-scores: 2.9 ± 0.8 vs. 2.7 ± 0.9) were quite similar. There was slightly less overweight (10.3 vs. 15.4%) and more extremely obese subjects (35.1 vs. 28.3%) in this sub-group. The mean number of medical complications was 2.5 ± 1.6 (range: 0 to 8), and 4.3 ± 2.1 when adding non-medical ones. The repartition of the subjects according to the number of complications they present is displayed in Table 2. Almost 90% of the children are affected by at least one medical co-morbidity, without gender difference.

**Table 2:** **Prevalence of medical and non-medical complications and their correlation with BMI z-score, in a sub-group of 174 subjects**

Prevalence of complications in %	Number of complications					Corr coeff with BMI z-score
**N=**	**0**	**≥1**	**1**	**2**	**≥3**	
**Orthopedic**	32.2	67.8	32.2	28.7	6.9	0.129
**Cardiovascular**	48.3	51.7	28.2	15.5	8.0	0.152*
**Metabolic**	59.8	40.2	25.9	10.9	3.4	0.090
**Subtotal**	**10.4**	**89.6**	**19.5**	**23.0**	**47.1**	0.206**
**Non-medical**	14.4	85.6	26.4	29.9	29.3	0.076
**Total**	**2.3**	**97.7**	**5.2**	**13.8**	**78.7**	0.200**

Corr coeff: correlation coefficient using Pearson correlation between BMI z-score and each complication.

*p < .05, **p < .01.

## Discussion

A careful evaluation of subjects with body weight excess, presented to a pediatric obesity clinic, revealed that both medical and non-medical complications affecting psychological well being are very frequent. Some, but not all of them increased with weight status. We categorized those complications as follows: orthopedic, cardiovascular, metabolic and non-medical.

Many complications have been reported in obese children, but few of them investigated the prevalence of common orthopedic conditions [14–16], representing the second most frequent complication, after the psychological one, found in our cohort. More than 50% of subjects have at least one of those conditions, similarly to previous results found for a similar population [16]. This is an important finding as physical activity may be limited due to pain or mechanical limitations [15, 17], and therefore may possibly impact on treatment’ success. Flat foot incidence in obese children ranges from 5 to 28%, mirroring our results [18–20]. Prevalence of musculoskeletal and lower limb valgus alignment complications vary depending on the studies: a previous large cohort study has shown that their prevalence is greater in overweight than in normal weight children (complaints: 18.3% vs. 4.8%; valgus: 11% vs. 3.2%) [15]. Two other small studies reported a higher rate of genu valgum in obese children (rate 50 and 55%) which confirm the difference between normal and overweight subjects [14, 16]. Hyperlordosis has been less well studied, but one report focusing on obese children showed a similar prevalence [16]. Interestingly, in our cohort, the prevalence of those complications seems to be more frequent in younger children, but it may simply reflect the fact that they were also the most obese. Indeed, after age adjustment, those conditions increased with weight status.

Cardiovascular diseases risk factors are widely studied in this population, as their numbers are related to the severity of atherosclerotic changes [21]. In our cohort study, we found that 23% of subjects had either systolic or diastolic hypertension, confirming previous studies (15 to 52%) [22–25]. The magnitudes of blood pressure z-scores were proportional to the degree of adiposity, as already documented [22, 24, 26], and the risk to present systolic HTN in the obese and extremely obese groups were increased by 2.5 and almost five fold, respectively, compared to the overweight group. HTN was not correlated to family hypertension. Furthermore 40% of children had dyslipidemia; however abnormal TG level, often described to be elevated in obese subjects [26], was very rare in our cohort without any clear explanation. The prevalence of abnormal lipids in the obese groups was similar than in the literature [26]. However they were lower than the one found in overweight subjects, contradicting preceding studies [22, 27]. However, it is difficult to compare those two groups, as only around 25% of overweight subjects were screened, against 40% of the obese and extremely obese children. This low rate was due to the fact that some of them had already been screened before or missed the blood test appointment. Furthermore, when an overweight child had no family history of cardiovascular disease, we were less prone to perform such exam at the beginning of the follow-up. On the contrary to HTN and orthopedic complications, lipids disturbances were not dependent of BMI, except for HDL-C, but were rather correlated to positive family history of dyslipidemia, corroborating the genetic predisposition put forward by others [28–30]. In our study, the total number of CVD risk factors per subjects was correlated to family history of ischemic events, also suggesting the genetic component.

The increased prevalence of abnormal glucose metabolism or type 2 diabetes is debated in youth [31–34]. Its detection is widely performed in clinical settings; however, our study revealed that only 10% of the tested children had glucose metabolism disturbances, which confirms results found in previous publications [31, 35]. Insulin secretion anomalies were far more frequent but interestingly were not related to the severity of obesity but rather to the subject’s age, which isn’t in total accordance with published data [10, 36]. Children with African origin had less insulin anomalies than those from European countries, corroborating epidemiological data showing a low risk of diabetes in African subjects [37], contrary to African-American [38, 39]. It is interesting to note that acanthosis nigricans and insulin resistance were concomitant in only 45% of children. As already known [40], waist circumference was a good indicator of insulin disturbance.

Among the other complications, elevated TSH level was present in only 8% of children in our cohort. Others showed that elevated TSH level was found in 10 to 23% of obese children, as a consequence rather than a cause of obesity [41]. Since weight loss, as well as natural history, are associated with normalization of TSH [42–44], the question of the necessity of such screening must be raised, especially if clinical hypothyroidism is not suspect during the clinical examination.

Increased hepatic enzymes, such as ALAT, suggest a non-alcoholic fatty liver disease (NAFLD) also in children [45]. In our study, 7% of obese and 20% of extremely obese subjects had an increased level of ALAT, which stands between 6 to 25% found in the literature studying similar populations [46, 47]. In our cohort, the prevalence was higher in Asian subjects but this result must be taken with caution as we had only 11 Asian subjects. Indeed, the prevalence doesn’t seem to differ according to ethnic background [48]. This condition is important to detect, as it can lead to hepatic fibrosis and cirrhosis [49]. Such finding may be a strong motivator for the child and his family to change behaviors, as no treatment, other than weight management, has proven to be effective.

Finally, almost 80% of children complained about non-medical conditions affecting their well-being and quality of life, and it is by far the most frequent complication found in this population. Physical appearance and well-being are very important during growth and are often the main sources of motivation to seek medical attention. Interestingly, these complaints were independent of the real weight excess and affected both boys and girls. A recent review about quality of life revealed that obese children have impaired physical, social and emotional functioning compared to their lean counterparts [50]. These may lead to psychological distress, and should be screened and addressed, as they may impact treatment success and psychosocial development.

The strengths of this study were the wide age range and the large percentage of children studied in the unique centre dealing with obese children in the area. However, we can’t exclude a selection bias, as the children attending our care center may be the most affected by their condition, making generalization difficult.

The limitations encountered with this cohort were first, that blood tests were probably performed more early in overweight subjects with a higher risk of co-morbidities, which may falsely increase the prevalence of complications in this group. Secondly, as the pubertal status was missing in the majority of the subjects, we couldn’t adapt the cut-off point for insulin level. We choose the limit at 15 *μU/ml*, corresponding to pubertal stage I, as the majority of the subjects for whom we had the data were at this stage. Third, we didn’t evaluate the quality of life using a validated questionnaire, due to setting limitation. And finally, missing data may introduce a bias in the relative prevalence of each complication in our population. To address this question, we selected a sub-group of children for whom data were complete. Results showed that non-medical complications were the most frequent, followed by orthopedic, cardiovascular and finally metabolic. In total, almost all the children suffered of at least one of those co-morbidities.

## Conclusions

As a conclusion, a large majority of children and adolescents with body weight excess seeking medical advice are suffering from a variety of complications. Psychosocial difficulties should not be underestimated as the related complaints were the most frequently reported and were not related to the magnitude of the obesity. All the co-morbidities should be actively screened as they are known to have an impact on future health. Pediatricians and general practitioners have a major role in this screening as they can improve quality of care by treating these complications as soon as possible. Such findings can also be a potential source of motivation to stabilized or reduce weight gain. Finally, the biggest challenge would be the prevention of excessive weight gain in the general population, as the prevalence of the majority of the co-morbidities was directly dependant on the weight status.

## Electronic supplementary material

Additional file 1: **Child and Adolescent Department.** (DOCX 108 KB)
