# Supplementary material for: Medical and non-medical complications among children and adolescents with excessive body weight
Source: BMC Pediatr. 2014 Sep 14;14:232. doi: 10.1186/1471-2431-14-232 (PMC4168248; doi:10.1186/1471-2431-14-232)
Supplement: Supplementary file 1 — Additional file 1: Child and Adolescent Department. (DOCX 108 KB) [file 12887_2014_1152_MOESM1_ESM.docx]

Child and Adolescent Department

**First visit**

| Date : | |  | Age : |  |
| --- | --- | --- | --- | --- |
| Pediatrician : | |  |  |  |
| Referred by : | |  |  |  |
| Seen with : | Mother, father, other | |  |  |

**History of weight gain and past treatments:**

**Family context:**

|  | Mother : | Father: |
| --- | --- | --- |
| Origins : |  |  |
| Maternal language : |  |  |
| Marital status : |  |  |
| Child main home  : |  |  |
| Formation level : |  |  |
| Profession / activity rate : |  |  |
| Weight : |  |  |
| Height : |  |  |

**Attitude and motivation of the family during the visit:**

|  |
| --- |

**Family medical history:**

|  | **Obesity** | **Diabetes mellitus** | **Hypertension** | **Dyslipidemia** | **Cardio-vascular diseases** |
| --- | --- | --- | --- | --- | --- |
| Mother |  |  |  |  |  |
| Father |  |  |  |  |  |
| Maternal grand-mother |  |  |  |  |  |
| Maternal grand-father |  |  |  |  |  |
| Paternal grand-mother |  |  |  |  |  |
| Paternal grand-father |  |  |  |  |  |
| Brother |  |  |  |  |  |
| Sister |  |  |  |  |  |

**Personal history:**

| Gestational age at birth : | | | |  |  |  |  |  |  |  |  |
| --- | --- | --- | --- | --- | --- | --- | --- | --- | --- | --- | --- |
|  | | | |  | | | | | |  | |
| Gestational diabetes : | yes | no | | | | | | | | | |
|  | | |  | | | | | | |  | |
| Birth weight : Birth height : | | |  | |  | | | | | | |
|  | | |  | | | | | | |  | |
| Breast feeding : exclusive | | | mixed | | | | | | |  | |
|  | | |  | | | | | | |  | |
| General development: | | |  | | | | | | |  | |
| Age of walking : | | | Language : | | | | | | |  | |
|  | | |  | | | | | | |  | |
| Pubertal development: | | |  | | | | | | |  | |
| Menarche’s age: | | |  | | | | | | | Menarche’s age for mother  : | |
| Cycle regularity: | | |  | | | | | | | Dysmenorrheal : | |
|  | | |  | | | | | | |  | |
| Hospitalizations : | | | Diagnostic : | | | | | | |  | |
|  | | | When : | | | | | | |  | |
|  | | |  | | | | | | |  | |
| Diseases / surgical interventions / accidents : | | | | | | |  | | | | |
| Allergies : | | |  | | | | | | | | |
| Medicaments/contraception : | | |  | | | | | | | | |
| Cigarette smoking/alcohol/drugs : | | |  | | | | | | | | |
| Other : | | |  | | | | | | | | |
| Other therapy (physio-ergo-psycho) : | | | | | |  | | | | | |
|  | | | | | |  | | |  | | |
| School : performance, integration, bullying, other | | | | | | | |  |  | | |
|  | | |  | | | | | | | |  |
|  | | |  | | | | | | | |  |

**Systematic anamneses:**

| Dyspnea and/or cough during physical activity |
| --- |
| Headaches |
| Articular pain |
| Constipation |
| Concentration difficulty |

| Sleep : | snoring |  | | |  | | |
| --- | --- | --- | --- | --- | --- | --- | --- |
|  | apnea |  | | |  | | |
|  | agitation |  | | |  | | |
|  | somnolence during the day  pattern (delayed onset or early or frequent awakenings) | | |  |  | | |
|  | Quantity: | Week days: | | | Week-end : | | |
|  |  |  |  |  |  |  |  |
|  |  |  |  |  |  |  |  |
|  |  |  |  |  |  |  |  |
| Depressiveness / poor emotional feelings:  Preoccupation with physical appearance:  Self-esteem: | | | | | |  |  |
|  | | |  | | | |  |

**Alimentation:**

| Breakfast : |  |
| --- | --- |
| Snack during the morning : |  |
| Lunch : |  |
| Snack during the afternoon : |  |
| Dinner : |  |
| Drinks : |  |
| Snacking : |  |
| Eating disorder : | |

**Screen viewing:**

| Duration : Week days: | Week-end : |  |
| --- | --- | --- |
|  |  |  |

Type of screen:

**Physical activity:**

| Very active : | | Moderately active: | | Little active : | |
| --- | --- | --- | --- | --- | --- |
|  | | |  |  | |
| Way to school : walking, public transportation, car, other | | |  | Duration : | |
|  |  |  |  |  |  |
|  | | |  | |  |
| Sports at school: | | | Duration: |  | |
| Sports outside school : | | | Duration: |  | |
|  | | |  |  | |
|  | | |  |  | |
|  | | |  |  | |
